# Polytechnic engineering mathematics: assessing its relevance to the productivity of industries in Uganda

**DOI:** 10.1186/s40594-017-0078-z

**Published:** 2017-09-15

**Authors:** Peter J. Jehopio, Ronald Wesonga

**Affiliations:** School of Statitics and Planning, Makerere University, Kampala, Uganda

## Abstract

**Background:**

The main objective of the study was to examine the relevance of engineering mathematics to the emerging industries. The level of abstraction, the standard of rigor, and the depth of theoretical treatment are necessary skills expected of a graduate engineering technician to be derived from mathematical knowledge. The question of whether these skills are imparted to benefit emerging economies still remains a big area of investigation. This study investigated the relevancy of engineering mathematics to the benefit of local industry in a developing economy, Uganda.

**Results:**

There was a significant difference between the mathematics being taught to the engineering technical students and the mathematics relevant to the engineering technical work in the industries (*p* value < 0.05). Implying that the mathematics taught to engineering technical students, though relevant, is not in the form of what the industries require. The use, practicability, depth, rigor, modernity, relevancy, and usefulness of the taught mathematics were all statistically different from the desired mathematical skills for the industry. Moreover, the extent of use of the engineering mathematics did not differ between the required and taught and among the civil, electrical, and mechanical branches of engineering.

**Conclusions:**

The mathematics syllabi for polytechnic institutes should be developed to directly support innovations and efficiency in the local industries. Therefore, a close collaboration between the polytechnic colleges and the local industries should be facilitated to achieve a sustainable industrial.

**Electronic supplementary material:**

The online version of this article (10.1186/s40594-017-0078-z) contains supplementary material, which is available to authorized users.

## Introduction

The study examined the relevance to the local industry of the engineering mathematics in the emerging economies (Zill et al. 2011). Identification of the required quality of engineering mathematics in the academic programs essential to the local industry has not been established leading to an apparent deviation from what is required for local industrial development. Clearly, a major aim in teaching mathematics to the engineering technical students is to provide and equip the learners with knowledge and skills, in particular when they eventually settle to work as engineering technicians. Yet industry might only welcome that which it may practically value as applicable, relevant, and useful and may only entertain the suitable elements of the mathematics. Moreover, the level of abstraction, the standard of rigor, and the depth of theoretical treatment that might be characteristics of the mathematics taught may not at all be called for in the industries. In which case, it should be considered that there exists a mismatch between the pedagogic philosophy of the mathematics educators at the polytechnics on the one hand and, on the other hand, the apparent utilitarian requirements of the engineering technicians working in industry (Duffy 2010; Grewal 1996).

With respect to relevance to industry, while the mathematical view of the engineering technicians working in industry could significantly differ from that of the mathematics educators, there could exist a difference among the mathematical views of the civil, electrical, and mechanical engineering technicians working in industry (Markes 2006). Similarly, the views of the civil, electrical, and mechanical engineering mathematics educators might also differ. Further, on the question of the relevance of the mathematics taught to the needs of the industries, it is deemed in the study that there exists a difference in view of those students who have had and those who have not had practical industrial experience.

### Industries in which engineering technicians work

The industrial sector in Uganda may be described as facilities and equipment used for producing, processing or assembling goods, and encompassing activities such as manufacturing, quarrying, and mining including oil and gas extraction, construction, and the utility sub-sectors (Marios et al. 2014). The major industries in Uganda consist of manufacturing, constructing, mining, and quarrying electricity, gas, and water (Uganda Bureau of Statistics 2007). In comparison with industrialized countries, Uganda’s industry is mostly small scale (84%) in which about 50% of the firms employ 10 persons or less. The medium- to large-scale manufacturing sector is relatively smaller but has substantial establishments in sugar, beer, cigarettes, cotton fabrics, gunny bags, fishnets, and cement. The industrial sector is characterized by low capacity utilization, estimated at 50% of installed capacity (Dorosh and Thurlow 2014). In recent years, the telecommunication sector has blossomed, mainly characterized by an involvement of multinational companies which include MTN Uganda, Airtel, Uganda Telecom, Africell Uganda, Smile Telecom, K2 Telecom, Smart Telecom, and Vodafone Uganda, with total subscription of over 20 million (Hawthorne et al. 2016; Ward and Zheng 2016).

### The polytechnic curriculum

The polytechnic curriculum in Uganda aims at training students to become engineering technicians, with necessary competency to exercise technical judgment and assume personal responsibilities for duties in the engineering technical field (Eluk 1994). The engineering technician is required to be able to understand the reasons and purposes of the operations for which s/he is responsible perform highly technical duties of an established or novel character and to carry out technical and managerial responsibility either independently or under the direction of qualified engineers and scientists. Besides, an engineering technician must have the personal ability of leadership and logical thinking (Hemlin et al. 2014; Khairullina et al. 2016). Mathematics at the polytechnic in Uganda is mainly taught through lectures, demonstrations, and project works. The content meant to be covered in 156 h encompasses number systems, algebra and matrices, trigonometric functions, hyperbolic functions, calculus, vectors, McLaurin theorem, ordinary and partial differential equations, harmonic analysis, Laplace transforms, and statistics (Stroud and Booth 2013).

### Research questions and hypotheses

Diametrically, this study was undertaken in an attempt to answer the following key questions (Archer and Davison 2008), taking polytechnics in Uganda as a case study with a view of generalization to the developing countries. These include the following: (i) Is the mathematics being taught to the engineering technical students at the polytechnics relevant to the industries? (ii) Is the level of abstraction, the standard of rigor, the depth of theoretical treatment in the mathematics taught to the engineering technical students at the polytechnic overemphasized? (iii) Do the civil, electrical, and mechanical engineering technicians working in industry require basically the same branches of mathematics? (iv) Does exposure to practical industrial experience aid engineering technical students to better prepare them for work in industry? and (v) Would close collaboration between the polytechnics and the human resource personnel in industry be a significant necessity in ensuring the relevance to industry of the mathematics topics taught to the engineering technical students? Table 1 presents eight hypotheses developed and tested in this study.

**Table 1:** Study hypotheses

Null hypothesis	Statement of the hypothesis
H_01_:	There is no difference between the mathematics being taught to the engineering technical students and the mathematics relevant to the engineering technical work in the industries with respect to the extent to which mathematics is being put to use in the industries in Uganda
H_02_:	There is no difference between the mathematics being taught to the engineering technical students and the mathematics relevant to the engineering technical work in the industries with respect to practicability, applicability, depth, rigour, modernity, relevance to the industries in Uganda
H_03_:	There is no difference between the mathematics being taught to the engineering technical students and the mathematics relevant to the engineering technical work in the industries with regards to usefulness to industries in Uganda, of the specific mathematics topics taught to the Engineering technical students
H_04_:	There is no difference between the mathematics being taught to the engineering technical students and the mathematics relevant to the engineering technical work in the industries with regards to relevance to the industries in Uganda in general.
H_05_:	There is no difference in the mathematics taught to the: civil, electrical and mechanical engineering technical students with regards to the extent to which mathematics is in use in the industries in Uganda
H_06_:	There is no difference in the mathematics taught to the: civil, electrical and mechanical engineering technical students with respect to, practicability, applicability, depth, rigour, modernity, relevance to the industries.
H_07_:	There is no difference in the mathematics taught to the: civil, electrical and mechanical engineering technical students with regards to usefulness to industries in Uganda, of the specific mathematics topics taught to the engineering technical students.
H_08_:	There is no difference in the mathematics taught to the: civil, electrical and mechanical engineering technical students with regards to relevance to the industries in Uganda of specific topics taught.

### Aspects of science technology engineering and mathematics

Several studies have tackled various issues related to engineering students’ capability in using mathematics to solve engineering or real life problems in workplace arena, especially right after their graduation (Hoyles et al. 2002; Kent and Noss 2002). Solving an engineering problem requires a thorough comprehension of the mathematical connectedness of the components of the problem and of the mathematical nature of the solution to be provided. Accordingly, the concept of mathematical competency was coined and moved in many investigations and considerations for engineering education. Mathematical competency refers to individuals’ capabilities to apply mathematics in situations where mathematics can play a fundamental role (Niss 2003). A set of eight mathematical competencies required by engineering students, thinking mathematically, reasoning mathematically, problem handling, modeling mathematically, representing mathematically, communicating mathematically, symbolism and formalism language, and using aids and tools, was articulated (Niss 2003). Consideration of the mathematical set implies that desired mathematical competencies of engineers and engineering lecturers should be explicitly considered in the outcomes of engineering mathematics courses (Firouzian et al. 2013).

The undergraduate engineering relies heavily on mathematics (Darlington and Bowyer 2016). In engineering, mathematics is the language used to communicate parameters and models and optimize solutions (House and Street 2004). Mathematical skills and knowledge are a critical factor in the success of engineering students (Berry et al. 2014; Virtanen et al. 2014). Success in engineering is highly dependent on student ability to connect mathematics to engineering (Loch and Lamborn 2016). It is beneficial for engineering students to acquire practical experiences with real-world relevance (Bourne 2014). Experts on engineering education emphasize that efficient learning environments should have real-world relevance and encourage meaningful reflection on authentic tasks (Herrington et al. 2014). The mathematics required for engineering degrees generally centers on calculus and, importantly, modeling (Alpers et al. 2014). Thus, engineering students should also “possess a conception of mathematics as problem solving rather than (also possible) as simply a list of algorithms”(Craig 2013). However, mathematics can be useful, particularly in areas such as product design (Davies et al. 2015). For structural engineering, geometry is important (Kent and Noss 2002). In pure mathematics, basic topics such as numbers, formulae, algebraic and differential equations, vectors, and tensors are vital to engineering (Garg et al. 1998).

Numerous studies demonstrate that engineering students struggle with a wide range of areas of mathematics, including algebra, matrices, logarithms, analytic skills, trigonometry, calculus, proof, logic, modeling, and problem-solving (Bernstein 2005; Garg et al. 1998; Shatz 2016). A study by Faulkner et al. (2016) found out that students’ mathematical competencies had decreased. A number of surveys of engineering lecturing staff established that many of them were pessimistic about their students’ mathematical abilities and believed that their students were mathematically weaker than previous cohorts (Wandel et al. 2015); (Sarwadi and Shahrill 2014). Indeed, mathematics in engineering problem is not new. The outcry regarding the “Engineering Problem” spans countries worldwide, including Sweden and South Africa (Engelbrecht et al. 2015; Onwubolu and Babu 2013), Croatia (Flegg 2015; Jukić Matić and Dahl 2014), Finland (Havola et al. 2011), and Australia (Henderson and Broadbridge 2009).

Acquired mathematical competencies of engineering students are not in line with the required mathematical competencies of engineers at the workplace (Firouzian et al. 2014). Subsequently, this study sought to establish the relevance of the mathematics taught to engineering technical students at polytechnics to local industries in Uganda.

## Methods

### Data sources and sampling

The study was conducted in Uganda with a central focus on polytechnic engineering mathematics. To achieve the study objectives, stratified cluster sampling was employed with the sample clustered into three groups comprising of students, educators, and employers in the industry. These groups of respondents were selected because they are directly associated with engineering technical education, either as products or producers of engineering technicians in Uganda. Table 1 shows the sample distribution.

A random sampling approach was employed throughout the selection process of the respondents. The respondents were selected from the industries in three municipalities, including Kampala City Council Authority, Jinja Municipality, and Mbale Municipality, which constitute at least 90% of the industries in Uganda. A total sample of 305 was distributed as shown in Table 1 with 126 (41.3%) educators, 123 (40.3%) industry personnel, and 56 (18.4%) students.

### Data collection and management

A questionnaire was developed and used for data collection (Additional file 1). There were four sections in the questionnaire: section A contained one item for identification only. Here, each respondent answering the questionnaire was classified under only one of the groups described in Table 1. Section B of the questionnaire contained eight items that elicited the extent to which mathematics was in use in the identified industry. Section C comprised ten items to collect data on practicability, applicability, depth, rigor, modernity, and relevance to the industries in Uganda of the mathematics taught to engineering technical students in Uganda polytechnics**.** The last section D consisted 16 items that examined the usefulness to industries in Uganda of specific mathematics topics taught to the engineering technical students at polytechnics in Uganda.

To facilitate quantification of the data, the construction of the responses was based on Likert-scale (Rossouw et al. 2011). The scoring of the responses was done by assigning to “Strongly Agree, Agree, Undecided, Disagree, and Strongly Disagree” the scores 5, 4, 3, 2, and 1, respectively, for a positive answer to a positive item and the scores 1, 2, 3, 4, and 5, respectively, for a negative answer to a negative item. The scores for each question were then summed for the sections B, C, and D of the questionnaire. Finally, the overall score that is the sum of the scores obtained in the sections B, C, and D for each subject was worked out. The sum of the scores for each individual respondent represented the positivity of the awareness, the concern or the consideration, or the perception or the experience of the subject. After the scoring of the responses, in order to facilitate comparisons of the group scores, arithmetic means and corresponding standard deviations were computed.

The reliability of the questionnaire was ascertained by first conducting a pre-test of the questionnaire. After the pre-test, the ambiguities and the inconsistencies in the initial questionnaire were revised to generate the final questionnaire. Moreover, the final questionnaire was not administered to the respondents that had initially participated in the pre-test. Over 98% of the questions were pre-coded, and the Cronbach alpha reliability score of 0.8, which is in the acceptable good range, was derived from all the items in the questionnaire.

### Data analysis

To test the hypotheses, we employed analysis of variance (ANOVA). This was due to the nature of data and several assumptions, including normally distributed dependent variable, independently drawn, and homogeneity of variances (Iversen and Norpoth 1987; Larson 2008). Comparison of F observed and F critical was done. Where the relationship was significant, the study located the precise source of the differences by performing pairwise comparisons between the means of the groups using the Tukey HSD technique.

Other techniques used in the analysis of data included the Fisher’s least significant difference (LSD) (Meier 2006; Williams and Abdi 2010), in case the number of respondents in the sample groups was equal. In case the number of respondents in the sample groups was not equal, the Fisher’s protected test was performed (Shaffer 1995). Specifically, to test hypothesis H_03_, the two-tailed Students’ (*t*) test for independent groups was used. This test was employed because the number of respondents in each of the independent groups concerned was small, that is less than 30, and the fact that the respondents had been selected through a random process ensured the t-distribution. All tests were conducted at *α* = 0.05 significance level. Whenever a null hypothesis was rejected, the conclusion was that the observed difference among the groups was statistically significant.

## Results

The results of the study are presented, beginning with sample distributions and group scores in Table 2. The table shows the computed coefficient of variation among groups and by the type of construct, section B through D. Of the 340 respondents targeted, 305 responded, giving a response rate of 90%. To facilitate comparisons, the group scores and coefficient of relative variation (CV) were computed for each group and are expressed as percentages.

**Table 2:** Sample description and group coefficient of variation

Group	Description	Sample (*n*)	Percentage (%)	Section score CV (%)			
				B	C	D	Overall
MATE	Mathematics engineering technician educators	29	9.5	14.1	8.3	7.1	7.6
CETE	Civil engineering technician educators	29	9.5	29.6	14.7	17.6	16.9
EETE	Electrical engineering technician educators	32	10.5	14.1	8.5	13.1	8.8
METE	Mechanical engineering technician educators	36	11.8	14.6	9.9	13.6	7.2
CETI	Civil engineering technicians working in industry	37	12.1	27.4	11.1	23.5	17.3
EETI	Electrical engineering technicians working in industry	46	15.1	16.4	9.5	18.9	12.7
METI	Mechanical engineering technicians working in industry	40	13.1	18.5	8.6	16.8	12
SHIT	Students who have had industrial training	28	9.2	8.9	8.4	5.9	5.4
SNIT	Students who have not had industrial training	28	9.2	14.9	9.1	12.9	8.3

Note: B, C, and D refer to sections in the questionnaire. Section B contained eight questions that elicited the extent to which mathematics was in use in the identified industry. Section C comprised of ten questions on mathematics practicability, applicability, depth, rigor, modernity, and relevance to the industries. Section D consisted 16 questions that examined the usefulness of mathematics topics to industries

Considering the CVs in section B, groups of respondents were ranked in the order in which mathematics was in use based on section B items of the questionnaire. Accordingly, the group consisting of students who had industrial training (SHIT, CV = 8.9) was ranked first, followed by the group for the mathematics engineering technician educators (METE, CV = 14.1), and then the group for the electrical engineering technician educators (EETE, CV = 14.1) while the group civil engineering technician educators (CETE, CV = 29.6) ranked last. Details of the assessment of section B based on CVs for other groups are presented in Table 1.

Similarly, the coefficients of relative variation found in section C of the Table 2 show that the group for mathematics engineering technician educators (METE, CV = 8.3) ranked first while the group civil engineering technician educators (CETE; CV = 14.7) ranked last on aspects related to group C regarding practicability, applicability, acceptability of the depth, the rigor, and the modernity, and relevance to the industries in Uganda of the mathematics taught to the engineering technical students. The coefficients of relative variation found in section D of the Table 2 revealed that students who have had industrial training (SHIT, CV = 5.9) ranked first whereas civil engineering technicians working in industry (CETI, CV = 23.5) ranked last on aspects to do with whether specific topics were important for engineering technical work in the industries in Uganda.

Overall, regarding to the relevance of the mathematics taught, the group consisting of students who had industrial training (SHIT, CV = 5.4) ranked first while civil engineering technicians working in industry (CETI, CV = 17.3) ranked last.

### Analysis based on testing hypotheses, H_01_ through H_08_

The eight hypotheses were assessed based on their aggregated group scores for each construct of the questionnaire. Table 3 shows the ANOVA tests based on the F-distribution statistics.

**Table 3:** Significance differences between groups using analysis of variation

Null (H_0_)	Source of variation	SS	df	MS	F-statistic
H_01_	Between groups	372.11	6	62.02	1.81
	Within groups	8277.49	242	34.20	
	Total	8649.60	248		
H_02_	Between groups	625.85	6	104.31	7.16
	Within groups	3525.36	242	14.57	
	Total	4152.20	248		
H_03_	Between groups	14,024.32	6	2337.55	28.77
	Within groups	19,662.31	242	81.25	
	Total	33,687.63	248		
H_04_	Between groups	32.847.11	6	5474.52	30.20
	Within groups	43,865.13	242	181.26	
	Total	76,712.24	248		
H_05_	Between groups	34.12	2	17.06	0.47
	Within groups	4349.05	120	36.24	
	Total	4383.17	122		
H_06_	Between groups	30.71	2	25.35	2.33
	Within groups	1304.65	120	10.87	
	Total	1355.35	122		
H_07_	Between groups	2360.43	2	1180.21	7.32
	Within groups	19,353.70	120	161.28	
	Total	21,714.12	122		
H_08_	Between groups	3577.50	2	1788.75	6.71
	Within groups	32,009.93	120	266.75	
	Total	35,587.43	122		

F-statistic stands for the quotient MS (between groups)/MS (within groups)

*SS* sum squares, *df* degrees of freedom, *MS* SS/df for the particular source of variation

The findings presented in Table 3 were used to draw conclusions for the eight hypotheses of the study using the ANOVA tests. Their different interpretations are thus presented below. An attempt was made to establish the source(s) of the significant differences using the Tukey HSD test, wherever identified. The acronyms to identify pairs of significant group differences were defined in Table 2.

H_01_: There is no difference between the mathematics being taught to the engineering technical students and the mathematics relevant to the engineering technical work in the industries *with respect to the extent to which mathematics is being put to use in the industries in Uganda*

The value for the F-statistic was 1.81, while its corresponding tabulated value at the significance level *α* = 0.05 was 2.10. The decision was not to reject the null hypothesis. Thus, the conclusion that there is no statistically significant difference between the mathematics being taught to the engineering technical students and the mathematics relevant to the engineering technical work in the industries with respect to the extent to which mathematics is being put to use in the industries in Uganda.

H_02_: There is no difference between the mathematics being taught to the engineering technical students and the mathematics relevant to the engineering technical work in the industries *with respect to, practicability, applicability, depth, rigour, modernity, relevance to the industries in Uganda*

The value for the F-statistic was 7.16, while its corresponding tabulated value at the significance level *α* = 0.05 was 2.10. The decision was to reject the null hypothesis. Thus, the conclusion that there is a statistically significant difference between the mathematics being taught to the engineering technical students and the mathematics relevant to the engineering technical work in the industries with respect to practicability, applicability, depth, rigor, modernity, and relevance to the industries in Uganda. Further analysis showed that the observed significance was mainly due to differences between the following pairs of the groups: (MATE, EETE), (MATE, METE), (MATE, CETI), (MATE, EETI), (MATE, METI), (CETE, CETI), (CETE, EETI), (METE, CETI), and (METE, METI).

H_03_: There is no difference between the mathematics being taught to the engineering technical students and the mathematics relevant to the engineering technical work in the industries *with regards to usefulness to industries in Uganda, of the specific mathematics topics taught to the Engineering technical students*

The value for the *F* statistic was 28.77, while its corresponding tabulated value at the significance level *α* = 0.05 was 2.10. The decision was to reject the null hypothesis. Thus, the conclusion that there is a statistically significant difference between the mathematics being taught to the engineering technical students and the mathematics relevant to the engineering technical work in the industries with regard to usefulness to industries in Uganda of the specific mathematics topics taught to the engineering technical students.

Further analysis showed that the observed difference was mainly due to the following pairs of the groups: (MATE, EETE), (MATE, METE), (MATE, CETI), (MATE, EETI), (MATE, METI), (CETE, CETI), (CETE, EETI), (CETE, METI), (EETE, CETI), (EETE, EETI), (EETE, METI), (METE, CETI), (METE, EETI), (METE, METI), (CETI, EETI), and (CETI, METI).

H_04_: There is no difference between the mathematics being taught to the engineering technical students and the mathematics relevant to the engineering technical work in the industries *with regards to relevance to the industries in Uganda in general.*

The value for the F-statistic was 30.20, while its corresponding tabulated value at the significance level *α* = 0.05 was 2.10. The decision was to reject the null hypothesis. Thus, the conclusion that there is a statistically significant difference between the mathematics being taught to the engineering technical students and the mathematics relevant to the engineering technical work in the industries with regard to relevance to the industries in Uganda in general.

Further analysis showed that the source of the statistical significance observed was due to the following pairs of the groups: (EETE), (CETI), (METI); (CETE, CETI); (CETE, EETI); (CETE, METI); (EETE, EETI); (EETE, METI); (METE, CETI); (METE, EETI); (METE, METI); (CETI, EETI) and (CETI, METI).

H_05_: There is no difference in the mathematics taught to the: civil, electrical and mechanical engineering technical students *with regards to the extent to which mathematics is in use in the industries in Uganda*

The value for the F-statistic was 0.47, while its corresponding tabulated value at the significance level *α* = 0.05 was 3.00. The decision was not to reject the null hypothesis. Thus, the conclusion that there is no statistically significant difference in the mathematics taught to the civil, the electrical, and the mechanical engineering technical students with regard to the extent to which mathematics is in use in the industries in Uganda.

H_06_: There is no difference in the mathematics taught to the: civil, electrical and mechanical engineering technical students *with respect to, practicability, applicability, depth, rigour, modernity, relevance to the industries.*

The value for the F-statistic was 2.33, while its corresponding tabulated value at the significance level *α* = 0.05 was 3.00. The decision was not to reject the null hypothesis. Thus, the conclusion that there is no statistically significant difference in the mathematics taught to the civil, the electrical, and the mechanical engineering technical students with respect to practicability, applicability, depth, rigor, modernity, and relevance to the industries.

H_07_: There is no difference in the mathematics taught to the: civil, electrical and mechanical engineering technical students *with regards to usefulness to industries in Uganda, of the specific mathematics topics taught to the engineering technical students.*

The value for the F-statistic was 7.32, while its corresponding tabulated value at the significance level *α* = 0.05 was 3.00. The decision was to reject the null hypothesis. Thus, the conclusion that there exists a statistically significant difference in the mathematics taught to the civil, the electrical, and the mechanical engineering technical students with regard to usefulness to industries in Uganda of the specific mathematics topics taught to the engineering technical students.

Further analysis established that the source of the statistical significance observed was due to the differences between the pairs of the groups: civil engineering technicians working in industries against electrical engineering technicians working in industries (CETI, EETI) and civil engineering technicians working in industries against mechanical engineering technicians working in industries (CETI, METI).

H_08_: There is no difference in the mathematics taught to the: civil, electrical and mechanical engineering technical students *with regards to relevance to the industries in Uganda of specific topics taught.*

The observed value of the F-statistic was 6.71, while its corresponding tabulated value at the significance level *α* = 0.05 was 3.00. Thus, the null hypothesis was rejected, indicating the existence of a statistically significant difference in the mathematics taught to the civil, the electrical, and the mechanical engineering technical students with regard to relevance to the industries in Uganda of specific topics taught. Further analysis showed that the source of the statistical significance observed was due to the difference between the pairs of the groups: civil engineering technicians working in industries against electrical engineering technicians working in industries (CETI, EETI) and civil engineering technicians working in industries against mechanical engineering technicians working in industries (CETI, METI).

## Discussions

The purpose of the study was to examine the relevance to the industries in Uganda of the mathematics taught to engineering technical students at polytechnics in Uganda. Our findings point to the fact that developing countries like Uganda need to develop engineering mathematics curricula that directly respond to the necessary and sufficient requirement for mathematics in their polytechnic institutes.

### Findings based on hypotheses H_01_ to H_04_

Our findings show that there is no significant difference between mathematics being taught to the engineering technical students at polytechnics and that which is required in the engineering technical work by the industries in Uganda. This may be interpreted to imply that the mathematics being taught at the polytechnics is actually far wider than the needs of the industries and not that the needs of the industries and the mathematics taught are exactly equitable. Here, one may be tempted to believe that the industries are operating outdated machines and probably less scientific. Advisably, in an attempt to satisfy the needs of the industries in Uganda, the mathematics course offered at the polytechnics should not be curtailed to the needs of any industries operating less scientifically, but rather should be tailored for a normal, up to capacity, and modern industry. As Welch and Freebody (2002) observed, the challenge falls on the technological educators to produce “technically qualified, literate graduates who not only meet the needs of the industry today but will be able to grow to meet the needs of industry and society tomorrow.”

The preceding argument should indeed prove acceptable, given that in testing of the hypothesis H_02_, it was concluded that, with respect to practicability, applicability, depth, rigor, modernity, and relevance to the industries in Uganda, of the mathematics taught, a significant difference exists between the mathematics being taught to engineering technical students and that which is actually called for in engineering technical work in the industries. This was further confirmed in testing of the hypothesis H_03_ where it was seen that, with regard to usefulness to industries in Uganda, of the specific mathematics topics taught, there exists a significant difference between the mathematics taught and that which is actually called for by the industries in Uganda (Mativo et al. 2013).

### Findings based on hypotheses H_05_ to H_08_

Findings show that there is no significant difference in the mathematics taught to the civil, the electrical, and the mechanical engineering technical students at the polytechnics. And also, with respect to practicability, applicability, depth, rigor, modernity, and relevance to the industries in Uganda (Tseng et al. 2013), there was no significant difference in the mathematics taught. The acceptance of the hypotheses H_05_ and H_06_ suggests further that besides general considerations, each of the civil, the electrical, and mechanical, industries in Uganda do require mathematics to a high extent (Sanders 2008).

The hypotheses H_07_ and H_08_ were however rejected. Thus, it was concluded that there was no significant difference in the mathematics taught to the civil, the electrical, and the mechanical engineering technical students at the polytechnics. Obviously, there are specific areas where the interests of different groups of the engineering technicians differ (Liebenberg and Mathews 2012). Advisably, in designing mathematics courses for engineering technicians, there is a need for core mathematics topics, useful to all, and other elective mathematics topics so as to cater for the needs of the individual groups of engineering technicians more effectively (National Research Council 2011).

Here, four general observations are discussed. Firstly, the extent to which mathematics is in use, or required, by the industries in Uganda, is high. However, a clear disparity was observed between the perception of the engineering technicians working in the industries in Uganda and that of the engineering technicians’ educators at the polytechnics in Uganda. The likely explanation of the observed disparity was the fact that, here, the respondents were drawn from varied and specialized industrial backgrounds, whose individual mathematics interests were, as such, varied and stereotyped, unlike at the polytechnics where both the educators and the students are a “compact space” whose ideas, likely, tend toward a confluence of theoretical considerations (Brand 2003).

Secondly, contrary to popular belief that “engineering technical students are by nature not interested in any mathematical depth”, our study revealed that sufficient depth in engineering technical mathematics may be safely explored where the depth is useful to engineering technical work (Atkin and Black 2005; Langen and Dekkers 2005). The outcome of the study also emphasized close collaboration between mathematics lecturers and personnel in industry as an important step toward the successful teaching of mathematics that would be relevant to industry (Council, T. A. and National Research Council 2001).

Thirdly, on the mathematics topics taught, there were specific areas where interests of engineering technicians differed, depending on group biases. Therefore, in designing mathematics course for engineering technicians, the core mathematics topics useful to all should be separated from elective mathematics topics so as to cater for the need of the individual groups of engineering technical students. Thus, for the mathematics taught to engineering technical students to remain relevant to the industry, the study recommends regular review and appraisal of the syllabi so that topics found necessary are introduced while those found wanting are discarded accordingly (Mills and Treagust 2003).

Lastly, the students who had practical industrial experience do perceive, rather more accurately, the relevance to the industries in Uganda (Klingbeil et al. 2005; Klingbeil et al. 2004). The findings particularly point to the fact that practical industrial experience is important in the training of engineering technicians. In effect, this implies that the idea of sandwich courses needs to be encouraged in the training of engineering technicians (Croft and Ward 2001).

## Limitations

The study employed a quantitative approach to collect the data. However, to create a balance of the multiplicity of respondents, a concurrent mixed methods approach would have been more suitable for this study as it would have brought out the opinions of the lecturers and management in the industry where the polytechnic college graduates are employed. Furthermore, this study does not claim inadequacy in skills of engineering technicians solely on mathematics knowledge and skills because mathematics is just one of the course units undertaken as a requirement for engineering graduates of polytechnic colleges. Nonetheless, findings derived from this study are representative of the target population and can be relied upon to draw important policy decisions for the development of mathematical skills necessary for the development of industries. This study anchors science, technology, engineering, and mathematics principles especially for the engineering technicians in the developing countries.

## Conclusions

In conclusion, the study demonstrated that mathematics taught to engineering technical students was relevant to the industries in Uganda. However, more studies are required to qualify the relevancy conclusion since industries in this part of the world are still far less developed than would demand for higher levels of mathematical knowledge and skills. This study recommends a close collaboration between the mathematics lecturers at the polytechnic colleges and the personnel in the industry so as to calibrate the necessary engineering mathematics (Schilling and Klamma 2010). To effect such collaboration, the polytechnics should facilitate lecturers to conduct visits to the industry so as to assess the engineering mathematics’ requirements as they constantly review its syllabi. Similarly, there should be a mandatory attachment of students to industry for practical hands-on experience at least once during their study period so as to relate mathematics knowledge to the industry. Likewise, industry should send their employees for refresher courses in mathematics at the polytechnics. The mathematics taught should be regularly reviewed and appraised to sustain its relevancy and promote employability of the right skilled staff in industry for higher productivity.

Sustainability of developing countries such as Uganda is hinged on industrial development that is subsequently reliant on the human resources and, more specifically, the engineering profession. Thus, the need for proper planning in developing required mathematical skills that directly benefit industrial development.

## Additional file

Additional file 1: Data collection questionnaire for the study. (DOCX 18 kb)

## Abbreviations

ANOVA: Analysis of variance
LDC: Least developed countries
LSD: Least significant difference
MSW: Mean sum of squares
SSW: Sum of squares within
