# Supplementary material for: Polytechnic engineering mathematics: assessing its relevance to the productivity of industries in Uganda
Source: Int J STEM Educ. 2017 Sep 15;4(1):16. doi: 10.1186/s40594-017-0078-z (PMC6310385; doi:10.1186/s40594-017-0078-z)
Supplement: Supplementary file 1 — Data collection questionnaire for the study. (DOCX 18 kb) [file 40594_2017_78_MOESM1_ESM.docx]

**APPENDIX**

**QUESTIONNAIRE USED IN THIS STUDY**

**QUESTIONNAIRE**

**Introduction:**

This questionnaire aims at gathering information that will be used to investigate the relevance to the industries in Uganda, of the mathematics taught to Engineering Technical Students at the Ugandan Polytechnic. The findings of this investigation will be used in designing engineering technician mathematics syllabuses that are consonant with the current and possibly the future needs of the industries in Uganda. Your response to this questionnaire will be very valuable.

**SECTION A**

In this section, tick only the one response that is true to you.

1. In which **one** of the following disciplines or categories do you fall?
2. Engineering Technician Educator **(Mathematician)** at Uganda Polytechnic
3. Engineering Technician Educator **(Civil Engineer)** at Uganda Polytechnic
4. Engineering Technician Educator **(Electrical Engineer)** at Uganda Polytechnic
5. Engineering Technician Educator **(Mechanical Engineer)** at Uganda Polytechnic
6. Engineering Technician **(Civil)** working in industry.
7. Engineering Technician **(Electrical)** working in industry.
8. Engineering Technician **(Mechanical)** working in industry.
9. Student, at Uganda Polytechnic:
10. who **has had** (adequate) practical industrial experience.
11. who **has not had** (adequate) practical industrial experience.

**SECTION B**

In this section there are five possible responses to each question. The possible responses are:

1. Strongly Agree (SA);
2. Agree (A);
3. Undecided (UD);
4. Disagree (D);
5. Strongly Disagree (SD).

Please, to each of the following questions clearly tick one and only one of the responses that best represent your experience (if you are an Educator, give the response that best represents your pedagogic (educational) philosophy).

1. There is no much mathematical

thinking in industry. (SA) (A) (UD) (DA) (SDA)

1. There is much mathematical

reasoning in industry. (SA) (A) (UD) (DA) (SDA)

1. The nature of work being carried

out in industry does not call

for much depth in mathematics (SA) (A) (UD) (DA) (SDA)

1. In industry, mathematics aids

in planning constructions and

designs, and in evaluating

projects (SA) (A) (UD) (DA) (SDA)

1. In industry, mathematics is not

an aid in arriving at desirable

accuracy and precision (SA) (A) (UD) (DA) (SDA)

1. Mathematics is being employed

as a medium of communicating

engineering information in

industry (SA) (A) (UD) (DA) (SDA)

1. Mathematics aids in learning

about new methods, new processes

and new equipment (SA) (A) (UD) (DA) (SDA)

1. In industry, mathematics

aids in Quality Control (SA) (A) (UD) (DA) (SDA)

**SECTION C**

In this section there are five possible responses to each question. The possible responses are:

1. Strongly Agree (SA)
2. Agree (A)
3. Undecided (UD)
4. Disagree (D)
5. Strongly Disagree (SD).

Please, to each of the following questions clearly tick one and only one of the responses that best represent your experience (if you are an Educator, give the response that best represents your pedagogic (educational) philosophy).

1. Engineering Technical Students

need be told the reason why

they should learn any branch

of mathematics (SA) (A) (UD) (DA) (SDA)

1. The mathematics taught to the

Engineering Technical Students

should be linked to the

immediate practical need only (SA) (A) (UD) (DA) (SDA)

1. The mathematics taught to the

Engineering Technical Students

Need not be linked to future

likely usefulness (SA) (A) (UD) (DA) (SDA)

1. Abstraction in the mathematics

taught to Engineering Technical

Students should be avoided at

all costs. (SA) (A) (UD) (DA) (SDA)

1. Sufficient depth in engineering

technical mathematics may be

explored, where the depth is

useful to engineering work (SA) (A) (UD) (DA) (SDA)

1. Rigorous treatment appears to be

completely fruitless in

engineering technical

mathematics (SA) (A) (UD) (DA) (SDA)

1. Engineering technical

mathematics should be kept

strictly applied (SA) (A) (UD) (DA) (SDA)

1. Computing, including computer

Science need be taught to

Engineering Technical Students (SA) (A) (UD) (DA) (SDA)

1. Computers and computer based

machinery are becoming

common in industry (SA) (A) (UD) (DA) (SDA)

1. There need exist close

collaboration between lectures of

mathematics at Uganda Polytechnic

Kyambogo and industrial

personnel (SA) (A) (UD) (DA) (SDA)

**SECTION D**

The following questions examine specific topics contained in the engineering technical mathematics syllabuses taught at Uganda Polytechnic Kyambogo; in relation to the mathematics in use in industries in Uganda. This time the possible responses are:

1. Very Useful, (VU);
2. Useful, (U);
3. Undecided, (UD);
4. Useless, (UL);
5. Very Useless, (VUL).

Please, to each of the following questions clearly tick one and only one of the responses that best represent your experience (if you are an Educator, give the response that best represents your pedagogic (educational) philosophy).

1. Complex numbers. (VU) (U) (UD) (UL) (VUL)
2. Vectors. (VU) (U) (UD) (UL) (VUL)
3. Trigonometric Functions. (VU) (U) (UD) (UL) (VUL)
4. Hyperbolic Functions. (VU) (U) (UD) (UL) (VUL)
5. Series (Maclaurins, etc.). (VU) (U) (UD) (UL) (VUL)
6. Calculus (differentiation). (VU) (U) (UD) (UL) (VUL)
7. Calculus (Integration). (VU) (U) (UD) (UL) (VUL)
8. Curves and Curve fitting (VU) (U) (UD) (UL) (VUL)
9. Ordinary differential equations (VU) (U) (UD) (UL) (VUL)
10. Partial differential equations (VU) (U) (UD) (UL) (VUL)
11. Laplace Transforms (VU) (U) (UD) (UL) (VUL)
12. Fourier Series (VU) (U) (UD) (UL) (VUL)
13. Matrices and determinants. (VU) (U) (UD) (UL) (VUL)
14. Numerical Analysis (VU) (U) (UD) (UL) (VUL)
15. Probability and Statistics. (VU) (U) (UD) (UL) (VUL)
16. Write down, if any, topic(s) that are not currently covered in the engineering technician mathematics at Uganda Polytechnic, but that need necessarily be included.

Please use the space provided below.

1. …………………………………………………………………………………..
2. …………………………………………………………………………………..

****************

THANK YOU.

****************
